# Reducing Bias in Estimates of Per Protocol Treatment Effects: A Secondary Analysis of a Randomized Clinical Trial

**DOI:** 10.1001/jamanetworkopen.2023.25907

**Published:** 2023-07-26

**Authors:** Stephen R. Cole, Jessie K. Edwards, Paul N. Zivich, Bonnie E. Shook-Sa, Michael G. Hudgens, Jeffrey S. A. Stringer

**Affiliations:** 1 Department of Epidemiology, Gillings School of Global Public Health, University of North Carolina, Chapel Hill; 2 Institute of Global Health and Infectious Diseases, School of Medicine, University of North Carolina, Chapel Hill; 3 Department of Biostatistics, Gillings School of Global Public Health, University of North Carolina, Chapel Hill; 4 Department of OB/GYN, School of Medicine, University of North Carolina, Chapel Hill

## Abstract

This secondary analysis of a randomized clinical trial evaluates ways of reducing bias in estimates of per protocol treatment effects.

## Introduction

Intent-to-treat effect estimates from randomized trials are influenced by participant adherence to the treatment protocol. When protocol adherence is imperfect, estimation of per-protocol effects is recommended.^1,2^ Frequently, per-protocol effects are estimated by censoring or excluding participants who deviate from a protocol (Table). This approach provides valid estimates if protocol deviations are noninformative (ie, completely at random within treatment groups).

**Table.** Examples of Recent Randomized Trials Reporting Unstandardized Per-Protocol Effects

Source^a^	Primary outcome	Treatment groups	Estimate (95% CI)	
			Intent-to-treat	Per-protocol
Goldenberg et al, 2022	Symptomatic recurrent venous thromboembolism by 1 y	Anticoagulant therapy of 6 wk (207 participants) or 3 mo (210 participants)	−1.1 (−5.2 to 2.3)	0.0 (−3.8 to 3.5)
Grillot et al, 2023	Successful tracheal intubation on first attempt	Neuromuscular blockers (575 participants) or remifentanil (575 participants)	−6.1 (−11.6 to −0.5)	−5.7 (−11.3 to −0.1)
Nyang’wa et al, 2022	Death, treatment failure or stop, loss to follow-up, or tuberculosis recurrence by 72 wk	Bedaquiline, pretomanid, linezolid, and moxifloxacin (151 participants) or standard of care (152 participants)	−30 (−46 to −14)	−9 (−22 to 4)
Reis et al, 2022	Hospitalization or emergency department visit due to COVID-19 by 28 d	Ivermectin for 3 d (679 participants) or placebo (679 participants)	0.90 (0.70 to 1.16)^b^	0.94 (0.67 to 1.35)^b^
Turkova et al, 2022	Treatment failure, loss to follow-up, or death by 72 wk	Standard first-line pediatric antituberculosis treatment for 16 (602 participants) or 24 (602 participants) wk	−0.6 (−2.3 to 1.4)	0 (−2.9 to 2.9)
Van der Vaart et al, 2022	Patient-reported improvement at 24 mo	Pessary treatment (218 participants) or surgery (222 participants)	−6.1 (−12.7 to ∞)^c^	−13.1 (−23.0 to ∞)^c^
Varghese et al, 2023	Death by 28 d, persistent complications at day 7, or persistent fever at day 5	Intravenous azithromycin and doxycycline (266 participants) or doxycycline (265 participants)	−13.3 (−21.6 to −5.1)	−15.0 (NA)

Abbreviation: NA, not available.

^a^ Further information about articles is available in the eAppendix in Supplement 2.

^b^ Reis et al provides a risk ratio; all other sources provide risk differences.

^c^ One-sided CIs.

However, protocol deviations can be informative, especially when deviations are caused by factors associated with risk for the outcome. Reporting per-protocol effects without accounting for the possible bias induced by censoring protocol deviations is analogous to reporting an observational study with no control for confounding. To minimize the potential for such bias, one can account for protocol deviations using a per-protocol estimator that standardizes for observed variables with inverse probability weights,^3^ generalized computation,^4^ or comparable approaches.

## Methods

In this secondary analysis of a randomized clinical trial, we illustrate how failing to account for informative protocol deviations can lead to biased estimates using data from a trial of 17 alpha-hydroxyprogesterone caproate (17P) to reduce preterm birth among HIV positive pregnant people. This study was approved by the local institutional review board, and participants provided written informed consent. The study followed the Consolidated Standards of Reporting Trials (CONSORT) reporting guideline (see Supplement 1 for further details). Data were analyzed with SAS version 9.4 (SAS Institute) in February 2023.

## Results

In the Improving Pregnancy Outcomes with Progesterone (IPOP) trial,^5^ 800 HIV-positive pregnant people were randomly assigned to weekly injections of 17P (399 participants) or placebo (401 participants) to reduce preterm birth (before 37 weeks) between 2018 and 2020. Nearly all participants (760 participants; 95%) adhered to a protocol allowing only 1 missed injection. The estimated intent-to-treat effect was a risk ratio (RR) of 1.00 (95% CI, 0.64-1.55). At randomization, 363 participants (45%) had a mid-trimester cervical length less than 4 cm, which was associated with a 2-fold risk of preterm birth.^6^

To illustrate the potential for bias arising from differential protocol deviations, suppose contrary to fact, cervical length was also a predictor of protocol deviation, such that 152 of 186 (81%) participants in the 17P group with a cervix less than 4 cm deviated from the protocol but only 95 of the remaining 614 (15%) participants deviated from the protocol. Under such a scenario, if we censor protocol deviants as did the studies in the Table, then the unstandardized per-protocol RR estimate comparing 17P vs placebo in the 553 uncensored participants is 0.74 (95% CI, 0.41-1.32), suggesting a lower risk of preterm birth in the 17P group, as the authors hypothesized. But the standardized per-protocol RR estimate, accounting for cervical length using inverse probability weights,^3^ is 1.02 (95% CI, 0.52-2.02). This standardized result is as expected because the estimated intent-to-treat RR was 1 and actual protocol deviations were minimal.

## Discussion

When protocol deviations are not completely at random within treatment groups, an unstandardized per-protocol effect estimator can be biased, even grossly, as shown in the example. The example enjoys all the benefits of counterexamples but has the limitations of being partially contrived and having restricted scope. Using postrandomization information on protocol adherence to censor participants requires accounting for possible induced bias. Standardized per-protocol effect estimators will generally be valid if the common causes of protocol deviations and outcomes are measured and accounted for appropriately.^1,2^ However, typical regression models fail to account for postrandomization variables appropriately even when a sufficient set of variables is measured.^1^ But standardization methods such as inverse probability weighting and generalized computation can provide valid per-protocol effect estimates when a sufficient set of variables is measured.^3,4^

Although there is 1 intent-to-treat effect (for the observed amount of adherence), there are as many per protocol effects as there are possible definitions of protocol deviation. In the 17P example previously mentioned, participants were classified as deviating from protocol when they missed more than 1 weekly injection, but protocol deviations could have been defined less stringently as, for example, more than 3 missed injections. In conclusion, we recommend randomized trials report the estimated intent-to-treat effect, and when protocol deviations occur, a series of standardized per-protocol effect estimates which vary the stringency of the definition of a protocol deviation.

## Notes

Supplement 1.
Trial Protocol

Supplement 2.
eReferences

Supplement 3.
Data Sharing Statement
