# Supplementary material for: Reducing Bias in Estimates of Per Protocol Treatment Effects: A Secondary Analysis of a Randomized Clinical Trial
Source: JAMA Netw Open. 2023 Jul 26;6(7):e2325907. doi: 10.1001/jamanetworkopen.2023.25907 (PMC10372700; doi:10.1001/jamanetworkopen.2023.25907)
Supplement: Supplement 1. — Trial Protocol [file jamanetwopen-e2325907-s001.pdf]

Improving Pregnancy Outcomes with Progesterone (IPOP): a Trial of 17-Hydroxyprogesterone Caproate to Reduce Preterm Birth Among Women Receiving Antiretroviral Therapy in Pregnancy

---

Protocol version 1.6
26 March 2020

Funded by:
Eunice Kennedy Shriver
National Institute of Child Health and Human Development
1R01HD087119

and

The Bill and Melinda Gates Foundation

Study Drug Provided by:
AMAG Pharmaceuticals, Inc

Protocol Co-Chairs:
Bellington Vwalika, MBChB, MSc, MMed
Jeffrey S. A. Stringer, MD

Trial Registration:
ClinicalTrials.gov
NCT03297216

NB: This protocol is organized and formatted according to the guidelines set forth by the University of Zambia Biomedical Research Ethics Committee (UNZA BREC)

Protocol Signature Page

Protocol Title: Improving Pregnancy Outcomes with Progesterone (IPOP): a Trial of 17-Hydroxyprogesterone Caproate to Reduce Preterm Birth Among Women Receiving Antiretroviral Therapy in Pregnancy

Short Title: IPOP

Protocol Version: 1.6

Version Date: 26 March 2020

I, the Investigator of Record, agree to conduct this study in full accordance with the provisions of this protocol. I will comply with the provisions of this protocol, all requirements regarding the obligations of clinical investigators as fully outlined in the International Conference on Harmonization (Section E6(R1) Good Clinical Practice), local regulatory requirements, and the Investigator’s Agreement, which I have also signed.

I have read and understand the information in the Investigator's Brochure(s), including the potential risks and side effects of the product under investigation, and will ensure that all staff assisting in the conduct of the study are informed about the obligations incurred by their contribution to the study.

Investigator of Record Name: ______________________________

Investigator of Record Signature: ______________________________

Date: ______________________________

TABLE OF CONTENTS

INVESTIGATORS AND SCIENTIFIC TEAM 4
ABBREVIATIONS AND ACRONYMS 5
PROTOCOL SUMMARY 6
TRIAL SCHEMA 7
1.0 Introduction 8
2.0 Statement of the Problem 8
3.0 Rationale 8
4.0 Background and Literature Review 9
5.0 Specific Objectives 11
5.1 Primary objective 11
5.2 Secondary objectives 12
5.3 Tertiary objectives 12
6.0 Study Outcomes 12
6.1 Primary outcomes 12
6.2 Secondary outcomes 12
6.3 Tertiary outcomes 12
7.0 Trial Methodology 13
7.1 Study design 13
7.2 Study sites and study population 13
7.3 Study intervention 14
7.4 Study drug pharmacological information 14
7.5 Study procedures 14
7.6 Biological specimen collection and testing 19
7.7 Quality control and quality assurance procedures 19
7.8 Data security and management 19
7.9 Statistical considerations 20
7.10 Dissemination of findings 23
8.0 Ethical Considerations 24
8.1 Informed consent 24
8.2 Potential risks to participants 24
8.3 Potential benefits to participants and others 25
8.4 Inclusion of children, sub-populations, and vulnerable populations 26
9.0 Timeline 26
10.0 Budget 26
11.0 Reference List 28

INVESTIGATORS AND SCIENTIFIC TEAM

Protocol Chairs:

Bellington Vwalika, MBChB, MMed, MSc
Professor and Chair
Department of Obstetrics and Gynaecology
University of Zambia School of Medicine
Lusaka, Zambia
Email: vwalikab@gmail.com

Jeffrey S. A. Stringer, MD, FACOG
Professor of Obstetrics and Gynecology
UNC School of Medicine
Campus Box 7570, Chapel Hill, NC 27599
Email: jeffrey_stringer@med.unc.edu

Investigator of Record (IOR):

Joan T. Price, MD, MPH
Research Assistant Professor
Department of Obstetrics and Gynecology
UNC Chapel Hill
Campus Box 7570, Chapel Hill, NC 27599
Email: joan_price@med.unc.edu

Co-Investigators:

Margaret Kasaro, MBChB, MS
UNC Global Projects - Zambia
348 Independence Avenue
Lusaka, Zambia
E-mail: margaret.kasaro@unclusaka.org

Stephen R. Cole, PhD
Professor
Department of Epidemiology
UNC Chapel Hill
McGavran-Greenberg Hall 2105E
Campus Box 7435, Chapel Hill, NC 27599
Email: cole@unc.edu

Benjamin H. Chi, MD, MSc
Professor of Obstetrics and Gynecology
UNC School of Medicine
Campus Box 7570, Chapel Hill, NC 27599
Email: bchi@med.unc.edu

Elizabeth M. Stringer, MD, MSc
Associate Professor of Obstetrics and Gynecology
UNC School of Medicine
Campus Box 7570, Chapel Hill, NC 27599
Email: Elizabeth_stringer@med.unc.edu

Scientific Advisory Committee Members

Lynne M. Mofenson, MD
Elizabeth Glaser Pediatric AIDS Foundation
1140 Connecticut Ave, NW (Suite 200)
Washington, DC 20036
Email: mofensonl@mail.nih.gov

Robert L. Goldenberg, MD
Professor of Obstetrics and Gynecology
Columbia University
161 Fort Washington Avenue
New York, NY 10032
Email: rlg88@columbia.edu

Elwyn Chomba, MBChB
Professor Emerita
University of Zambia School of Medicine
Lusaka, Zambia
Email: echomba@zamnet.zm

Dwight J. Rouse, MD, MSPH
Professor of Obstetrics and Gynecology
Brown University
101 Dudley Street
Providence, RI 02912
Email: DRouse@Wihri.org

ABBREVIATIONS AND ACRONYMS

17P	17-alpha hydroxyprogesterone caproate
AE	Adverse event
ANC	Antenatal care
ART	Antiretroviral therapy
CI	Confidence interval
DSMB	Data safety and monitoring board
EDD	Estimated due date
GAPPS/PPB	Global Alliance for Prematurity Prevention / Preventing Preterm Birth
HIV	Human immunodeficiency virus
IM	Intramuscular
IPOP	Improving Pregnancy Outcomes with Progesterone (this trial)
KDC	Kamwala District Clinic
NICHD	National Institute of Child Health and Human Development
PI	Principal investigator
PTB	Preterm birth
RCT	Randomized controlled trial
RR	Risk ratio
SAE	Severe adverse event
SOP	Standard operating procedure
UNC	University of North Carolina
UNZA	University of Zambia
UTH	University Teaching Hospital
ZEPRS	Zambian Electronic Perinatal Record System

PROTOCOL SUMMARY

Background: Preterm birth (PTB) complicates one in six pregnancies in Zambia. Many infants who are born prematurely die as neonates while others may face lifelong disability. Maternal HIV is also highly prevalent in Zambia and associated with a 50% increase in PTB risk. Antiretroviral therapy (ART) in pregnancy appears to increase the risk of PTB even more. Injectable 17-hydroxyprogesterone caproate (17P) reduces the risk of PTB among women with a prior preterm birth and is standard of care for this population in the United States. However, 17P has not been studied as PTB prophylaxis in African populations or among HIV-infected women.

Objectives: To assess the efficacy of 17P for preventing PTB among HIV-infected pregnant women receiving ART.

Design: Phase III, placebo-controlled, double-masked, randomized controlled trial (RCT).

Study Arms: Weekly intramuscular injection of 250mg 17P or indistinguishable placebo randomly allocated in a 1:1 ratio started between 16-24 weeks of gestation and administered weekly thereafter until 36 $^{6}/_{7}$ gestational weeks, stillbirth, or delivery, whichever is sooner.

Population: 800 pregnant HIV-infected women over the age of 18 who enter antenatal care (ANC) prior to 24 weeks of gestation. Liveborn infants will also be enrolled at birth.

Study Sites: University Teaching Hospital (UTH), Kamwala District Clinic (KDC), or another district clinic in Lusaka, Zambia.

Duration and Follow up: Enrollment will proceed over a period of approximately 40 months. Individual participants will be followed from enrollment (prior to 24 weeks of gestation) through 42 days postpartum.

Endpoints: The goal of this intervention is to improve the likelihood that a woman will deliver a live-born, full-term infant. Thus, the primary trial outcome is a composite comprising the following: (1) delivery prior to 37 weeks of gestation, and/or (2) stillbirth at any gestational age.

Relevance: More than 1.5 million HIV-infected women become pregnant each year worldwide. Approximately half have access to ART, but all are at increased risk of PTB. 17P is a promising and cost-effective intervention to prevent PTB that should be studied in this high-risk population. Given the prevalence of HIV infection as a PTB risk factor in sub-Saharan Africa and in many other settings, the potential public health benefit of this intervention is substantial. The effectiveness of 17P to prevent PTB among HIV-infected women needs to be studied in a randomized trial.

TRIAL SCHEMA

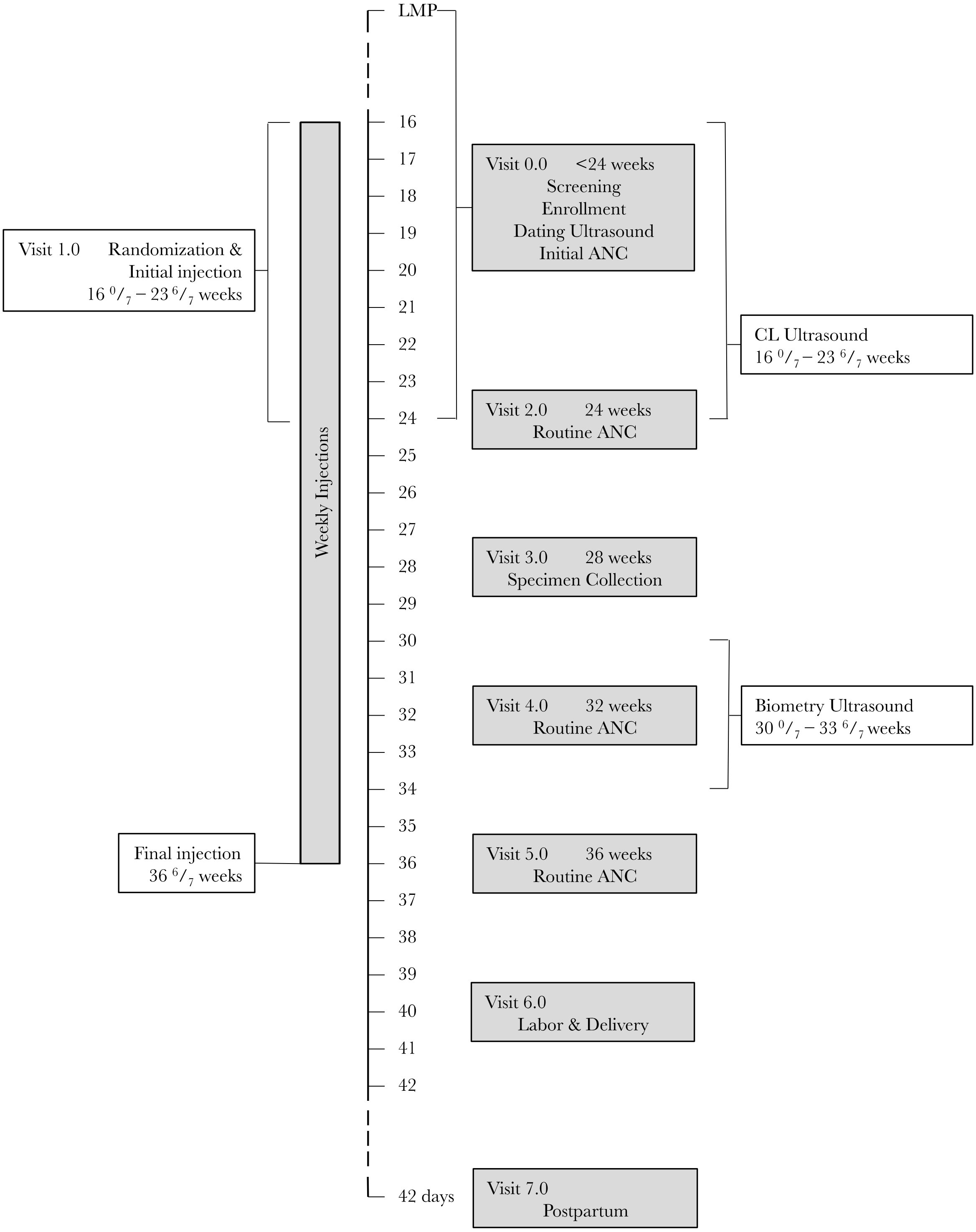

1.0 Introduction

In Zambia, PTB complicates one in six pregnancies.[1] Many infants who are born too soon die immediately while others face lifelong disability. Maternal HIV is also highly prevalent in Zambia and associated with a near doubling of PTB risk.[2,3] As access to combination ART has become available to an ever-increasing number of pregnant women around the world, perinatal HIV transmission rates have plummeted, but a new complication has emerged. Paradoxically, ART in pregnancy appears to increase the risk of PTB even beyond that attributable to HIV infection itself.[4]

Prenatal progestogens reduce the risk of PTB among women with evidence of cervical shortening or history of a prior spontaneous PTB.[5] Weekly 17P is FDA-approved and standard of care for this latter indication in the United States but has yet to be studied to reduce PTB among HIV-infected women. We hypothesize that the increased risk of PTB associated with HIV and ART may be amenable to 17P prophylaxis and propose a randomized trial to test this hypothesis.

IPOP is a phase III randomized, placebo-controlled, double-masked trial to assess (1) whether intramuscular 17P (started between 16-24 weeks of gestation and administered weekly thereafter until 36 $^{6}/_{7}$ gestational weeks, stillbirth, or delivery, whichever is sooner) will reduce the rate of PTB among HIV-infected pregnant women receiving ART; (2) whether women who start ART in pregnancy are at higher risk of PTB compared to those who enter care on ART initiated prior to conception; and (3) biological correlates of preterm birth and other adverse birth outcomes in the trial cohort. Approximately 800 women will be enrolled in Lusaka, Zambia from the University Teaching Hospital, the Kamwala District Clinic, or another district clinic.

2.0 Statement of the Problem

PTB is the most common cause of neonatal death worldwide. Preterm neonates who survive are at an increased risk of short- and long-term morbidity. Women who are HIV-infected have higher than usual chances of delivering their baby prematurely. Given high prevalence of both HIV and PTB in Zambia, the prevention of PTB in this high-risk group is a health care priority.

3.0 Rationale

More than 1.5 million HIV-infected women become pregnant each year. Approximately half have access to antiretroviral therapy (ART), but all are at increased risk of PTB. Injectable 17-alpha hydroxyprogesterone caproate (17P) is a promising, low-cost intervention to prevent PTB that should be studied in this population.

We propose to apply this known effective intervention to a new, exceedingly prevalent indication. Despite the abundance of progesterone PTB trials in the published literature,[6-18] none have targeted patients at risk of prematurity owing to HIV infection. Similarly, there are no 17P trial data from sub-Saharan Africa, a region that shoulders more PTB disease burden relative to its population size than anywhere else on earth.

Because HIV is so prevalent worldwide, this intervention – if proven effective – could have a substantial public health impact. UNAIDS estimates that 50% of HIV-infected women who become pregnant each year have access to combination ART.[35] We estimated a baseline risk of prematurity of 12.3%,[1] that HIV infection is associated with a relative risk (RR) of

prematurity of 1.8,[2] that receipt of ART is associated with a RR of prematurity of 2.2,[19] and that 17P could reduce the risk of prematurity by 38% (i.e. RR=0.62).[20] Under these assumptions, HIV and ART are responsible for an additional 184,500 preterm births each year worldwide, a number that will only increase as ART coverage continues to rise. With reasonable integration of 17P use into existing PMTCT programs, it is conceivable that the intervention could be delivered to 25% of HIV-infected women not receiving ART and 80% of those receiving ART. Under these assumptions, the 17P intervention would prevent 40,664 PTBs per year, and perhaps have the added benefit of enhancing ART adherence in pregnancy.

4.0 Background and Literature Review

4.1 Global Burden of Preterm Birth

An estimated 15 million babies are born prior to 37 weeks of gestation every year, one million of whom die as a direct result of their prematurity.[21] Over 60% of the world's preterm deliveries occur in Africa and South Asia; in sub-Saharan Africa the rate of preterm birth approaches 20%, compared to rates less than 10% in the Global North.[1] The survival of premature infants depends heavily upon where a baby is born: PTBs in low- and middle-income countries are much more likely to result in neonatal mortality due to unequal access to life-saving technologies and inadequate basic neonatal care. Beyond mortality, lifelong complications of prematurity include neurodevelopmental disorders (e.g. cerebral palsy), vision and hearing loss, learning impairment, and other chronic diseases.[22] Global funding and research has focused largely on developing new technologies to improve outcomes of the preterm infant, much of which is currently applicable only in North America and Europe. Of the strategies that have been studied for PTB prevention, few have proved effective, especially in low- and middle-income countries.[23] Low-cost, high-impact interventions are desperately needed to prevent PTB among those at highest risk for prematurity and its consequences.

4.2 HIV and Preterm Birth

Two large meta-analyses performed nearly two decades apart have each shown that HIV-infected women have higher rates of PTB compared to uninfected women.[2,3] The more recent analysis, which included 35 studies through 2014, reported a pooled risk ratio (RR) of PTB among HIV-infected women (not on ART) to be 1.5 times that of uninfected women (RR 1.50; 95% CI: 1.24, 1.82).[3] The strongest and most consistent effect was noted in sub-Saharan Africa, where some 85% of HIV-infected pregnant women reside.[24] HIV-infected women taking ART, with[25-27] or without[28-30] protease inhibitors, experience even higher rates of PTB when compared to HIV-infected, untreated women.[31-39]

4.3 Progesterone and Preterm Birth

Prenatal progesterone – which can be administered intramuscularly or vaginally – reduces the risk of PTB in women who have had a prior spontaneous PTB and in those with evidence of cervical shortening via mid-trimester sonography. Weekly 17P, administered via intramuscular injection, is FDA-approved and recommended as standard of care by the Society of Maternal Fetal Medicine to reduce the risk of PTB among women with a prior spontaneous PTB.[40] A 2013 Cochrane meta-analysis of progesterone to prevent PTB among women with a prior PTB aggregated data from 10 randomized trials that studied prenatal prophylaxis by the intramuscular (IM, n=4 studies), vaginal (n=5 studies), or oral (n=1 study)

route and estimate the risk ratio of birth prior to 37 weeks among women receiving active drug to be 0.55 (95% CI: 0.42, 0.74). When restricted to only those studies that provided IM drug (as we propose in this study), the aggregate RR was 0.62 (95% CI: 0.52, 0.75).[5]

4.4 Anti-inflammatory Properties of Progesterone

The initiation of labor – whether term or preterm – is an inflammatory event[20] and progesterone has potent anti-inflammatory properties.[20,41] Progesterone suppresses inflammatory cytokines and prostaglandins that induce uterine myocyte contraction, weaken the membranes, and promote cervical remodeling/ softening.[42-46] Progesterone receptor antagonists induce labor in animal models, an effect that can be reversed or delayed with add-back progesterone.[47,48] Declining concentrations of circulating progesterone and reduced progesterone activity are associated with preterm labor.[49] With supplementation, higher serum concentrations of progesterone have incremental protection against preterm labor.[50] HIV infection causes immune activation and inflammation, both systemically and in the lower genital tract.[51,52] While treatment with suppressive ART generally improves systemic inflammation, its effect on the lower genital tract is not nearly as consistent.[53-55] Indeed, HIV-infection is associated with bacterial vaginosis,[56-58] an alteration of the vaginal microbiome that predisposes to PTB[59,60] and that may not be resolved with ART.[61] Further, in the weeks following ART initiation, many women have increased shedding of viral pathogens, such as HSV and CMV in the vagina.[62,63]

Trials of progestogens to prevent PTB have used both intramuscular (IM)[9-12] and vaginal[64-68] drug. Although vaginal administration is common in the United States – especially for women with a shortened cervix in the mid-trimester – only the IM route is currently FDA approved. At least two other studies are piloting vaginal progesterone in HIV infected women. These include a Canadian trial that will randomize 40 women (NCT02400021) and a Zambian trial that will randomize 120 women (NCT02970552).

Specific biologic markers indicating increased risk of preterm delivery have been difficult to elucidate. While we suspect that biomarkers found in amniotic fluid, urine, cervical mucus and vaginal secretions, serum, and plasma may play a role in risk of PTL, PPROM and preterm delivery, few analytes have been identified that have clinical utility. Part of the difficulty lies in timing of collection in relation to gestational age and delivery. If a woman is already in PTL, then noting an increase in a specific metabolite is of little value in predicting PTL; however, identification of these analytes can increase our understanding of PTL pathophysiology. Thus, a more systematic approach to collection of possible biomarkers during routine antenatal care among women who are followed for their entire pregnancy and post-partum care can provide an enhanced understanding of which analytes may contribute to PTL, PPROM, and preterm delivery. [69,70]

4.5 Safety of Progesterone in Pregnancy

The human placenta produces increasingly large amounts of progesterone throughout pregnancy and 17P is a natural metabolite of endogenous progesterone. The concentrations of naturally occurring 17P exceed the pharmacologic doses proposed by this study.

4.5.1 Animal Studies

Studies of 17P in various species of pregnant non-human animals have demonstrated no adverse androgenic effects on developing fetuses. Certain progestestational compounds are

known to virilize female fetuses in both rat models and human studies.[71-74] However, in the first mouse model, assays of two nonandrogenic progestins – 17-alpha ethinyl-19-nortestosterone acetate (17-ENT) and 17P – demonstrated virilization of female fetuses only with 17-ENT but not with 17P exposure; male fetuses were not affected by either compound.[75] Even with administration of doses much higher than the equivalent human dose, 17P has shown no virilizing effects on female fetuses of rats, rabbits, or monkeys.[76-79]

Similarly, no teratogenic effects of 17P have been noted in numerous studies of pregnant animal models treated with 17P. In monkeys given 17P for threatened abortion in early pregnancy, surviving fetuses were all normal and healthy.[79] Two studies in mice evaluating the teratogenic potential of 17P administered doses up to 70 and 200 times the human equivalent. These high-doses of 17P during organogenesis did not increase the frequency of congenital abnormalities, fetal limb morphogenesis, or growth.[80,81]

4.5.2 Human Studies

Several controlled clinical trials[9,68,82,83] and epidemiological studies[84,85] have demonstrated the safety of 17P in human pregnancy. In randomized trials of 17P for the prevention of PTB, no significant risk of adverse birth outcomes has been noted in women with progesterone exposure compared to placebo. While some trials have reported a statistically non-significant increased risk of stillbirth and/or miscarriage,[6,9,17] others show no trend,[15] and others report a nonsignificant reduction in stillbirth with 17P.[8] A individual participant data meta-analysis of 13 trials including 3,768 women showed a relative risk of adverse perinatal outcome of 1.1 (95% CI 0.97–1.4) among women receiving 17P compared to placebo, and concluded that treatment with 17P does not have an effect on adverse perinatal outcomes.[86]

Two extensive reviews argue that no evidence exists that exposure to 17P in utero induces genital malformations in female fetuses.[85,87] In addition, the rates of non-genital malformations in fetuses exposed to 17P compared to unexposed fetuses have been similar in numerous large clinical trials and population reports. In one, 988 infants exposed to exogenous progesterone or 17P were compared to a matched cohort of unexposed infants in the United States; similar rates of all congenital malformations were reported between the two cohorts.[84] A prospective study comparing 1,608 newborns exposed to progestogens (either oral medroxyprogesterone caproate or 17P, or both) in the first trimester of pregnancy and 1,146 unexposed newborns found nearly equal rates of both major and all malformations between the two groups.[88]

4.5.3 Side effects of Intramuscular 17P in Pregnancy

Side effects of 17P may include headache, breast tenderness, nausea, cough and local irritation at the injection site.[89] The latter is the most commonly reported event, with the following specific symptoms: urticaria (12%), pain (35%), and swelling (17%).[9] Women with one or more contraindications to 17P that appear in the package insert will be excluded from the trial (see section 7.2)

5.0 Specific Objectives

5.1 Primary objective

The primary objective of this study is to evaluate, among HIV-infected pregnant women receiving ART, whether 17P will reduce the risk of the composite outcome (live birth prior to 37 weeks of gestation or stillbirth at any gestational age), relative to placebo.

5.2 Secondary objectives

- To assess, through a subgroup analysis, whether 17P will reduce the risk of the composite primary outcome among women either (a) newly initiating ART or (b) continuing ART that was started prior to conception
- To assess the effect of timing of ART initiation on risk of the composite primary outcome and its components by comparing women newly starting ART to those who initiated ART prior to conception (non-randomized comparison), and controlling for possible confounders (e.g., CD4 count, viral load, ART regimen).
- To assess the extent to which exclusion of provider-initiated PTB phenotypes modify (a) the observed efficacy (if any) of 17P to prevent the composite primary outcome and (b) its components and any association between timing of ART initiation and PTB.

5.3 Tertiary objectives

- To investigate the underlying biology of PTB and other adverse pregnancy outcomes with particular attention to inflammation, immune activation, and alterations of the vaginal microbiome.
- To determine the ability of prediction algorithms to correctly classify the gestational age of newborns at delivery

6.0 Study Outcomes

6.1 Primary outcomes

Our primary outcome will be a composite of live birth prior to 37 weeks of gestation or stillbirth at any gestational age.

6.2 Secondary outcomes

- Delivery prior to 34 weeks of gestation
- Delivery prior to 28 weeks of gestation
- Spontaneous delivery prior to 37 weeks of gestation
- Spontaneous delivery prior to 34 weeks of gestation
- Spontaneous delivery prior to 28 weeks of gestation
- Birth weight <10th percentile for gestational age
- Birth weight <3rd percentile for gestational age
- Mother-to-child HIV transmission by 6 weeks postpartum
- Cumulative incidences of the competing risks of stillbirth and PTB
- Neonatal and perinatal mortality rates
- Infant APGAR scores

6.3 Tertiary outcomes

- Birth weight <2500g
- Birth weight <1500g
- Measures of maternal markers of inflammation, immune activation, and alterations of the vaginal microbiome

- Measures of infant morbidity, such as ICU admission, supplemental oxygen requirement, and need for assisted ventilation
- Measures of maternal morbidity, such as pregnancy-induced hypertension, antepartum hemorrhage, preterm premature rupture of membranes, oligo- or polyhydramnios, chorioamnionitis, mode of delivery, and respiratory infection
- Adverse drug reactions
- Serious adverse events and events resulting in study product discontinuation

7.0 Trial Methodology

7.1 Study design

This will be a double-masked placebo-controlled randomized trial of 17P among HIV-infected women in Lusaka, Zambia. Participants will be randomly assigned to weekly intramuscular administration of either 17P or indistinguishable placebo to be commenced between 16 and 24 weeks of gestation. We will estimate effectiveness by comparing our primary outcome, a composite measure of delivery prior to 37 completed gestational weeks or stillbirth at any gestational age, between women who were randomized to active versus placebo in an intention-to-treat analysis.

7.2 Study sites and study population

This study will be conducted in the antenatal clinics of the University Teaching Hospital (UTH), Kamwala District Clinic in Lusaka, Zambia, or another district clinic.

Inclusion criteria:

1. 18 years of age or older
2. less than 24 $^{0}/_{7}$ weeks of gestation
3. viable intrauterine singleton pregnancy confirmed by ultrasound
4. antibody-confirmed HIV-1 infection
5. currently receiving ART or intending to commence ART in pregnancy
6. ability and willingness to provide written informed consent
7. intent to remain in current geographical area of residence for the duration of study
8. willing to adhere to weekly study visit schedule

Exclusion criteria:

1. confirmed prior spontaneous preterm birth
2. multiple gestation
3. known uterine anomaly
4. planned or *in situ* cervical cerclage
5. major fetal anomaly detected on screening ultrasound
6. indication for planned delivery prior to 37 weeks (e.g. prior classical cesarean)
7. threatened abortion, preterm labor, or ruptured membranes at time of enrollment
8. known allergy or medical comorbidity listed as a contraindication to 17P in the prescribing information
9. prior participation in the trial
10. any other condition (social or medical) which, in the opinion of the study staff, would make trial participation unsafe or complicate data interpretation.

7.3 Study intervention

Participants will be randomized 1:1 to one of two arms in the randomized controlled trial. The first arm (17P arm) will receive weekly injections of 250mg 17-hydroxyprogesterone caproate while the other arm (control arm) will receive indistinguishable placebo.

7.4 Study drug pharmacological information

Pharmacologic Category: Synthetic progestin

Chemistry: 17-hydroxyprogesterone caproate (250mg/mL) is a sterile solution of hydroxyprogesterone caproate in castor oil for injection. Micronized progesterone is active by oral and vaginal routes of administration. It is a clear, yellow, sterile, non-pyrogenic solution for intramuscular injection. It is an ester derivative soluble in ethanol, acetone or ether; slightly soluble in tea oil or castor oil; and insoluble in water.

Each 5mL multidose vial contains hydroxyprogesterone caproate USP, 250 mg/mL (25% w/v), in castor oil USP (28.6% w/v) and benzyl benzoate USP (46% w/v) with the preservative benzyl alcohol NF (2% w/v).

Mechanism of Action: Hydroxyprogesterone caproate is a synthetic progestin. The mechanism by which hydroxyprogesterone caproate reduces the risk of PTB is unknown. However, endogenous progesterone is a potent anti-inflammatory and is thought to prevent preterm birth by counteracting inflammatory processes that lead to cervical remodeling, amniotic membrane rupture, uterine contractions, and the initiation of parturition. Prenatal progestins, either micronized progesterone via vaginal administration or 17P via intramuscular administration, are standard of care and recommended for the prevention of preterm birth by the Society for Maternal Fetal Medicine for women who have had a prior spontaneous preterm birth or those with shortened cervical length detected on ultrasound.

Formulation: Intramuscular injection

Strength: 250mg (1mL of 250mg/mL concentrated solution)

7.5 Study procedures

7.5.1 Screening

Our recruitment activities will begin with community sensitization in the catchment area of the recruitment clinics to educate community members about the trial and encourage early presentation for ultrasound. This community and clinic sensitization may be underway prior to initiation of this project as a result of other programs in the catchment areas.

Trained study staff will conduct health talks at the study site, focusing on the importance of antenatal care and possible prevention of preterm or early delivery, with emphasis on study inclusion and exclusion criteria. Staff will provide interested women with additional information and referrals to the study clinic for eligibility assessment.

Potential participants will be identified at their first antenatal visit. Women may be enrolled any time prior to 24 $^{0}/_{7}$ gestational weeks. Following a prescreening consent process, a dating ultrasound will be conducted per standard of care to confirm pregnancy location, viability, number of fetuses, and, among women within the recruitment window, cervical length

according to the method described by Iams et al.[90] Those who meet preliminary eligibility criteria who are interested in participating will complete the study informed consent process in English, Nyanja, or Bemba, depending on their language preference. Trial participation will be offered to all HIV-infected pregnant women who are determined to meet the listed inclusion and exclusion criteria.

After consenting for participation, women will be administered a series of questionnaires that gather targeted information on relevant demographics, health and risk behaviors, medical and obstetrical history, medication use, stress, and nutrition. If not already done so in routine antenatal care, participants will undergo a physical exam, including testing for urinary tract infections and proteinuria, anemia, syphilis, and HIV (as outlined in TABLE 1), all of which are recommended as part of routine antenatal care. Treatment of HIV and any obstetric complications will also be according to standard practice in Zambia.

Blood, urine, and vaginal swabs will be collected as detailed in Table 1.

7.5.2 Randomization

Following final confirmation of eligibility, participants will undergo randomization into one of two study arms as soon as possible starting at $16\ ^{0}/_{7}$ gestational weeks and prior to 24 weeks. The trial will assign women 1:1 to active drug or placebo. A statistician from the UNC Center for AIDS Research Biostatistics Core not otherwise associated with the study will design the scheme using random permuted blocks.

7.5.3 Study product preparation and dispensation

Study product will be produced, labeled, and packaged by the manufacturer, AMAG Pharmaceuticals of Waltham, MA, USA, under the brand name of Makena®. Each package will have a unique identifying number produced from a random number generator that will be indexed in a secured database as either placebo or product. Packaged product will be shipped to each study site and dispensed by an on-site pharmacist. Participants will begin weekly administration of study product from the day of randomization (between $16\ ^{0}/_{7}$ and $23\ ^{6}/_{7}$ gestational weeks, inclusive) until $36\ ^{6}/_{7}$ gestational weeks, stillbirth or delivery, whichever is sooner. Trained study nurses will administer study product in the manner indicated on the package insert and in accordance with Good Clinical Practice.

7.5.4 Antenatal, intrapartum, and immediate postpartum study follow-up

The specific content of each study visit can be found in TABLE 1. Because the trial will be placebo controlled and treatment allocation double masked, all women will have identical visit schedules. Starting as early as possible between 16 and $23^{6}/_{7}$ gestational weeks, participants will receive a weekly 250 mg injection of study drug or matched placebo. At each visit, we will evaluate women for maternal side effects of IM progesterone, which are rare and generally mild, but include headache, breast tenderness, nausea, cough, and local irritation.

Routine antenatal and HIV care will be provided to all participants following Zambian national guidelines. Antenatal care visits are scheduled approximately every four weeks. Our study staff will work with participants to ensure follow-up visits coincide with scheduled antenatal visits as often as possible to reduce burden on participants with regards to time and travel.

At 24 and 28 weeks, blood, urine, and vaginal swabs will be collected as detailed in Table 1. At the time of delivery or soon thereafter, blood, urine, and vaginorectal swabs will be collected when possible. In addition, we will collect samples of the placenta, umbilical cord, and cord blood after delivery for various assessments. 4-5 drops of cord blood will be applied to designated filter paper within pre-printed circles. Newborn heel-prick samples will be collected 24–72 hours after birth or sooner if the newborn is discharged from hospital within 24 hours of delivery. At each weekly visit, oral and pharyngeal swabs will be collected for storage and future protocol-related testing.

The study team will obtain detailed information about the clinical management of the participant’s delivery, as well as the delivery outcome for both the mother and her infant from medical records when possible.

TABLE 1: Schedule of Evaluations

Visit Number	0.0	1.0†	2.0	3.0	4.0	5.0	6.0	7.0
Gestational Age (weeks)	<24	16-23[6]	24	28	32	36	Delivery^	42 days
**ADMINISTRATIVE/REGULATORY PROCEDURES**								
Informed consent	●							
Confirmation of eligibility		●						
Collection/review of locator info	●	●	●	●	●	●	●	●
Randomization		●						
**CLINICAL/BEHAVIORAL PROCEDURES**								
Ultrasound*	●	●			●			
Obstetrical history	●							
Medical history and clinical exam*	●		●		●	●	●	●
Concomitant medication assessment	●		●		●	●	●	●
Demographic history	●							
Behavioral and nutritional assessment	●							●
Maternal depression screen	●							●
Infant clinical assessment							●	●
**STUDY PRODUCT PROCEDURES**								
17P Adherence counseling		●	●	●	●	●		
Study drug injections‡		●	●	●	●	●		
Side effects assessment‡			●	●	●	●		
**LABORATORY PROCEDURES**								
Maternal HIV (rapid EIA)*	●							
Maternal pregnancy test*	●							
Maternal rapid syphilis *	●							
Maternal candida, gram stain*	●							
Maternal hemoglobin (HemoCue)*	●				●			
Maternal urinalysis (dip; culture if +)*	●				●			
Maternal viral load and T cell assays	●		●				●	

Visit Number<br>Gestational Age (weeks)	0.0<br><24	1.0†<br>16-23[6]	2.0<br>24	3.0<br>28	4.0<br>32	5.0<br>36	6.0<br>Delivery^	7.0<br>42 days
Placenta, membranes, cord blood storage							•	
Infant DBS for gestational age screening							•	
Infant HIV DNA PCR*								•
Maternal oral / pharyngeal swabs storage §			•	•	•	•	•	•
Vaginal-rectal swab storage	•		•	•			•	
Blood storage	•		•	•			•	
Urine storage	•		•	•			•	

† If screening occurs at or after 16 weeks of gestation, all procedures may be combined into a single screening/randomization visit

* Performed as standard antenatal care

‡ Occurs weekly through week 36, or until delivery, whichever occurs first

^ All procedures may not be completed for women who deliver off-hours or in a location without staff coverage.

§ Swabs will be collected at weekly injections visits and other visits as indicated. Collection may be deferred for participants who refuse or if necessary supplies are unavailable.

7.5.5 Postnatal study follow-up

Participants will be asked to return for one study visit during the postnatal period, at 42-days post-partum. At this visit, we will assess interval complications and/or mortality. Once again, participants will receive the routinely recommended screening and treatment. Infants’ weight, head circumference, length, mid-upper arm circumference, feeding, and general well-being will also be assessed. In the postnatal period, health education will be geared toward routine care of post-partum women and both preterm and term newborns.

At the 42-day postnatal visit, participants will again be screened for depression using the Edinburgh Postnatal Depression Scale. Those who exhibit a score consistent with signs of depression or suicidality will be immediately referred for further evaluation and treatment. Finally, oral / pharyngeal swabs will again be collected for storage and future testing.

7.5.6 Retention

Once a participant is enrolled in the trial, the study team will make reasonable efforts to retain her in follow-up to minimize bias associated with loss to follow-up. The study team will track retention rates and address any issues related to retention. Strategies may include:

- Thorough explanation of the study visit schedule and procedures during informed consent, and re-emphasis at each study visit.
- Encouragement of participants to discuss potential study participation with their husbands/partners and other influential family members before agreeing to enrol in the study.
- Collection of detailed locator information at screening, and review and updating of this information at each study visit.
- Use of appropriate and timely visit reminder mechanisms (including phone calls and text messages, if participants specifically agree).
- Follow-up on missed visits, including home or other off-site visits if agreed upon.
- Mobilization of trained outreach workers to complete in-person contact with participants at their homes and/or other locations.

7.5.7 High priority conditions

Samples collected via cord blood and heel prick for metabolic gestational age screening may reveal screen positive results for high priority conditions, such as congenital hypothyroidism, hemoglobinopathies, and medium-chain acyl-CoA dehydrogenase deficiency. These conditions have the potential for confirmatory testing and for intervention in the early stages of an infant's life. Upon obtaining these results, the family will be notified of the identified condition and study personnel will facilitate a referral for confirmatory testing and treatment.

If the findings from any other testing performed in real time or on stored specimens provide information that may be directly relevant to a participant's or her infant's health care, the study staff will make reasonable efforts to notify her of the results.

7.5.8 Safety monitoring

At each study visit, study staff will evaluate participants for social harms and adverse events (AEs) including adverse drug reactions or events related to venipuncture or 17P administration. A social harm will be defined as a non-medical untoward consequence of study participation, including: difficulties in personal relationships, stigma, or discrimination from family or community. An AE will be defined as any untoward medical occurrence in a study participant including any abnormal sign (e.g., abnormal physical exam or laboratory finding), symptom, or disease, temporally associated with the individual's participation in the research, whether or not considered related to participation in the research. In addition to events related to study laboratory data s and study product, we can expect that this population of HIV-infected pregnant women to experience adverse events unrelated to study procedures, including adverse obstetrical outcomes, opportunistic infections, side effects from ART or other medications, hospitalization, and death.

We expect events to be minimal, but all will be documented, assessed for seriousness / severity and relatedness, and carefully monitored. The severity of study-related adverse events and social harms will be graded using the National Institute of Health's Division of AIDS Table for Grading the Severity of Adult and Pediatric Adverse Events. We will also record information on all serious adverse events (SAEs) occurring in participants whether or not they are related to study participation or the study drug, including AEs that:

1. Result in hospital admission (unless hospitalization is preplanned, i.e. for delivery) or prolongation of existing hospitalization
2. Are immediately life-threatening, including drug reactions that necessitate discontinuation of study participation,
3. Cause significant, persistent, or permanent harm or disability, either physical or psychological,
4. Result in death, including fetal demise after 20 weeks of gestation
5. Are congenital anomalies/birth defects

Information on adverse events or social harms that are related to the study drug and all SAEs will be documented on study data forms and routinely reported to the Principal Investigator (PI) or designee. If the PI, co-investigators, or their designees determine that study-related adverse events are occurring at an unexpected rate, they will assess the need for and facilitate staff re-training, protocol amendment, or study cessation. Serious study-related adverse events will be reported to all regulatory bodies, the study sponsors, and the DSMB (see below) within 72 hours of site awareness. Other study-related AEs will be reported according to each ethics committees' individual guidelines.

We will constitute a Data Safety and Monitoring Board (DSMB) to periodically monitor trial performance, feasibility, and study outcomes. The group will comprise senior investigators with statistical, methodological, and topical expertise who are not otherwise involved with the study. Their first charge will be to review the study protocol and create a monitoring schedule and stopping guidelines, based on expected event rates. Other specific duties will include: (1) periodic assessments of recruitment, accrual, retention, and data quality; (2) Considering new external data as they come available, including scientific or therapeutic developments that may have an impact on participant safety or the ethics of the trial; (3) evaluation of scheduled interim analysis. Mortality outcomes will be reviewed by the DSMB during routine meetings. The DSMB will have authority to enjoin enrolment or stop the study altogether for reasons of patient safety. If an interim analysis were to show unequivocal benefit before follow-up were complete, we would support stopping the study and reprogramming remaining resources to make the intervention available to all participants.

The DSMB will also have a specific role in determining study feasibility. Prior to study commencement, the DSMB will develop clear indicators of trial feasibility and rules for determining that the trial has become futile based upon recruitment performance or inability to ascertain key endpoints. In the event that these criteria are met, we will close the trial.

7.6 Biological specimen collection and testing

All samples will be obtained from study participants by trained study staff according to approved standard operating procedures. All samples will be processed according to the assay manufacturers' specifications. Some specimens collected from patients in this protocol will be analyzed immediately per standard antenatal care guidelines. Others will be stored temporarily for later study-related analysis to identify inflammatory markers (e.g., chemokines, cytokines), metabolic analytes, proteins, hormones, transcripts, and infectious factors contributing to preterm birth and other adverse birth outcomes.

Samples will be primarily housed at the University Teaching Hospital, Department of Obstetrics and Gynecology. Use of stored specimens for testing that is not specifically designated in this protocol will require additional regulatory approval.

All laboratory testing will be performed by trained staff using standard operating procedures and according to specific assay manufacturers' specifications.

7.7 Quality control and quality assurance procedures

Standard Operating Procedures (SOPs) following manufacturer's protocols and detailing technical procedures involved (e.g. sample collection, processing and storage, assay procedures and how to interpret test results) will be developed and used by the study team. Tests will only be performed by certified laboratory personnel. Site coordinators will complete annual recertification. The certification process is an opportunity to ensure the highest specimen quality and standardize collection techniques.

7.8 Data security and management

Data collected from each participant will include sociodemographic information, relevant HIV and obstetrical history, longitudinal clinical details of current pregnancy and delivery, and results of laboratory specimen testing.

Study data management (e.g. data transmission, query resolution, etc.) will follow site data management SOPs. Study identification numbers will be used on all forms and

communications related to the study. A separate confidential register will link study identification numbers and participants' names. All data instruments and registers will be securely stored. Data will be entered into a custom built database and will be validated. Computers will be password protected and their access restricted to authorized study personnel. Backups of the data will be made on a weekly basis. Data may be transmitted electronically to the study investigators through secure cloud-based servers. Study information will not be released without written permission of the participant, except when necessary for monitoring by the relevant ethical committees or their designees.

Data will be disposed of following sponsor recommendations. Biological specimens will be destroyed as dictated by the University Teaching Hospital guidelines. Data will be disposed of after completion of the study following sponsor guidelines. At that time, electronic records, including linkage codes and identifiers, will be permanently deleted. Paper records will be shredded prior to disposal.

7.9 Statistical considerations

7.9.1 Trial outcomes

Because stillbirth and preterm birth are competing risks, the trial's primary outcome will be a composite of live birth prior to 37 weeks of gestation or stillbirth at any gestational age. We chose this outcome because it is (1) associated with substantial morbidity in Zambia, (2) incontrovertible, given that entry into the study is contingent upon early dating ultrasound, and (3) can be easily obtained even in the event of home delivery. Secondary outcomes will include: (a) delivery prior to 34 weeks of gestation; (b) delivery prior to 28 weeks of gestation; (c) spontaneous delivery prior to 37 weeks of gestation; (d) spontaneous delivery prior to 34 weeks of gestation; (e) spontaneous delivery prior to 28 weeks of gestation; (f) birth weight < 10th centile for gestational age; (g) birth weight < 3rd centile for gestational age; (h) mother-to-child HIV transmission by 6-weeks postpartum, and (i) cumulative incidences of the competing risks of stillbirth, spontaneous PTB, and non-spontaneous PTB. To ensure comparability with other prematurity trials,[6,50] we will also systematically assess neonatal and perinatal mortality rates; infant APGAR scores; infant birth weights; measures of infant morbidity such as ICU admission, supplemental O_2 requirement, and need for assisted ventilation; and measures of maternal morbidity, such as pregnancy-induced hypertension, antepartum hemorrhage, preterm premature rupture of membranes, oligo- or polyhydramnios, chorioamnionitis, and mode of delivery.

7.9.2 Sample size calculation: baseline event rate (p1)

We estimated the baseline rate of our primary outcome as follows:

- The ZEPRS system in Lusaka[91] estimates GA at birth from the last menstrual period (LMP), a method that, in our setting, consistently overestimates prematurity.[92] A study performed by our group of all public sector births in Lusaka between 2006 and 2010 found that 31,896 of 100,408 women (31.7%) delivered before their LMP-derived EGA reached 37 weeks completed gestation.[93]
- As of April 2017, 1116 women had been enrolled in the GAPPS Preventing Preterm Birth study and 497 had delivered. All enrollees have an early dating ultrasound at 20-24 weeks.[54] Based on reliable GA estimates from these ultrasounds, 18.1% of women overall delivered prior to 37 weeks.
- A large international modeling effort[1] published by *Blencowe et al* in 2010 estimated that 12.9% of infants in Zambia are born prior to 37 weeks of gestation. While this

effort aggregated hundreds of published studies to inform its models, no data were included from Zambia. Since the Zambia estimate is not consistent with our own measurements, we believe it may be artificially lowered by inclusion of country economic data in the models. (Zambia is categorized as “lower-middle income” economy, a threshold it achieves only by a tiny margin; data from neighboring Zimbabwe[94-96] are consistently reported in the 17-20% range.)

- Given the suspected underestimation of gestational age in the ZEPRS data, and the relatively small sample size in the GAPPS cohort, we applied the *Blencowe* figures to existing data from our site for these sample size calculations, arriving at an overall prematurity rate of 15.5% (arithmetic mean of Zambia country estimates and current PTB rate in GAPPS) for our calculation of the baseline event rate. We note that to underestimate the prematurity rate would be conservative vis-à-vis our sample size calculations.
- Available estimates for Zambia do not disaggregate prematurity risk by HIV status. We therefore queried ZEPRS,[91] which does capture birth weight completely and correctly. We used birth weight as a proxy for prematurity to estimate the relative risk of preterm birth by HIV status in our setting. An analysis of 211,318 public sector singleton births in Lusaka between 2006 and 2013 found that 17.2% of HIV-exposed infants weighed <2500g at delivery, compared to 11.8% unexposed infants. (Chibwesha et al, submitted for publication; TABLE 2).

TABLE 2: Risk of LBW among 211,318 singleton infants born to women of known HIV status in Lusaka

	<2500 gms*		<2000 gms		<1500 gms	
	N	%	N	%	N	%
**HIV - (78%)**	19828	11.8	7081	4.2	3878	2.3
**HIV + (22%)**	7956	17.2	3088	6.7	1720	3.7
**RR (95%CI)**		1.46 (1.43, 1.50)		1.59 (1.52, 1.65)		1.61 (1.53, 1.71)

**NB: Birth weight categories are not mutually exclusive*

- Although low birth weight is not a perfect proxy for prematurity (most notably, it does not distinguish growth restriction from preterm birth), it is the only outcome we have to estimate the preterm birth rates in HIV+ versus HIV- women in our setting. We used the antenatal HIV prevalence and relative risk of LBW delivery from TABLE 2, to estimate a rate of prematurity in HIV-infected women that would correspond to an overall rate of 15.5% (Goal Seek Function, MS Excel).
- Under these assumptions, we estimate that the prematurity rate would have to be **20.9%** among HIV-infected women and 14.0% among HIV-uninfected women to produce an overall rate of 15.5% in a setting of 22% maternal HIV prevalence at UTH. To be conservative, we have not assumed any additional prematurity risk conferred by receipt of ART.
- Finally, a study of more than 100,000 public sector deliveries performed by our group found a stillbirth rate of 21 per 1,000 live births.[93] Among the 21,816 HIV-infected parturients, the stillbirth rate was 27 per 1000 (95% CI: 25, 29). Because our composite primary outcome includes stillbirth, we add a 2.7% stillbirth rate to the 20.9% prematurity rate to arrive at a baseline event rate of 23.6%. Rounding to 2 significant figures, we have set the baseline event rate, **p1 = 0.24**.

7.9.3 Sample size calculation: effect size

A 2013 meta-analysis estimated the RR of preterm delivery for women using *any* progestogen to be 0.55 (95%CI: 0.42, 0.74).[5] For the purposes of our study, we will use the more conservative subtotal estimate from only the IM studies (FIGURE 1). This 0.62 RR translates to a **38% relative efficacy**.

FIGURE 1: Summary of IM progesterone trials in Cochrane[5] meta-analysis

Study or subgroup	Progesterone n/N	Placebo n/N
I Intramuscular		
Ibrahim 2010	8/25	13/25
Johnson 1975	2/18	12/25
Meis 2003	111/306	84/153
Saghafi 2011a	16/50	30/50
**Subtotal (95% CI)**	**399**	**253**

Total events: 137 (Progesterone), 139 (Placebo)
Heterogeneity: Tau² = 0.0; Chi² = 2.84, df = 3 (P = 0.42); I² =0.0%
Test for overall effect: Z = 5.11 (P < 0.00001)

Figure 2: Sample size per arm for 80% power

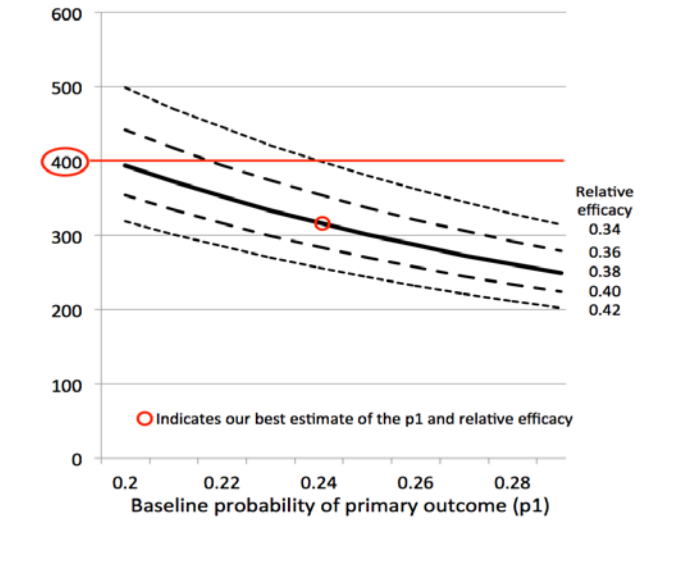

7.9.4 Trial sample size

In summary, we postulate that 17P will reduce the proportion of HIV-infected women experiencing the composite endpoint by 38% (from 24% to 15%). We used SAS 9.3 (SAS, Cary, NC) to determine that a trial with 325 patients per arm would have 80% power to detect this effect size (based on a 2-sided Fisher's exact test at the 0.05 significance level; FIGURE 2). To **allow for up to 15% follow-up loss** (although prior experience from our sites suggests it will be half this) and to account for uncertainty in our estimates of baseline event probability (p1) and relative efficacy of the intervention, we plan to enroll **400 women per arm** in the trial. As FIGURE 2 indicates, the study will have 80% power to demonstrate any combination of baseline event rate and relative efficacy that falls below the red horizontal line (after accounting for follow-up losses and any missing outcomes).

7.9.5 Stratification and randomization

We will stratify our randomization by timing of ART initiation (during current pregnancy / pre-pregnancy). Participants will be assigned with equal probability to one of the two treatment groups. A statistician not otherwise associated with the study will design the randomization scheme using random permuted blocks and taking into account the stratification scheme.

7.9.6 Analysis plan

Our **primary outcome** will be the composite outcome comprising delivery prior to 37 weeks completed gestation or stillbirth at any gestational age. At the time of enrollment, each participant will be assigned an estimated delivery date (EDD) by ultrasound biometry.[97-99]

Randomized women who deliver (1) a live infant prior to 37 weeks of gestation or (2) a stillborn infant at any gestational age will be categorized as having met the primary outcome.

The primary analysis will employ the intent-to-treat principle, wherein each mother-infant pair will be analyzed according to randomization assignment regardless of whether the mother actually complied with study procedures. The primary comparison between the two trial arms will be based on the estimated risk ratio of the primary outcome. The estimated risk of the primary outcome will be computed in each arm using the Kaplan-Meier method to adjust for loss-to-follow-up, where mother-infant pairs who drop out of the trial prior to the primary outcome or live-birth of full-term infant will be right censored at their last study visit. A *Z*-statistic will be constructed by taking the difference in the estimated log risks divided by the corresponding estimated standard error (using the Greenwood estimator). As a sensitivity analysis, a Cox proportional hazards model, stratified by timing of ART initiation (during current pregnancy / pre-pregnancy) will be fit to estimate the hazard ratio between randomization arms; the estimated hazard ratio is anticipated to be similar to the estimated risk ratio given that the primary outcome is relatively rare (<20%). In a secondary analysis, we will compare the risk of spontaneous preterm birth across randomized groups, treating provider-initiated preterm births as a competing risk and estimating the risk of the outcomes in each randomized group using the Aalen-Johannson method. To explore comparability with other published studies,[9] we will evaluate the effect of excluding events that occur prior to 20 weeks in a sensitivity analysis.

Subgroup analyses will assess whether the effect of 17P differed within levels of the randomization stratification factor. For timing of ART initiation, effect modification will be assessed by fitting Cox proportional hazards models that include as covariates randomization arm, the stratification factor, and a product term. A likelihood ratio test of the product term coefficient will be used to assess possible effect modification. To adjust for multiple comparisons, the likelihood ratio tests will be considered significant if $p < 0.05/2$. If the effect modification tests are significant, hazard ratios will be estimated separately within levels of the factor (e.g., hazard ratios of 17P versus placebo for (i) mothers on pre-pregnancy ART and (ii) mothers not on pre-pregnancy ART).

To assess the efficacy of 17P, a per-protocol analysis of the primary outcome will also be conducted that censors participants who fail to comply with their randomization assignment (defined here as missing two injections of study product in a row), and then reweights the data to account for possible informative censoring by noncompliance using inverse-probability (IP) weights.[100] We have previously used this and related methods to construct a consistent estimator of the per-protocol effect under the assumption that we have measured and correctly-adjusted for the common causes of noncompliance and the outcome.[101-103] The set of measured possible common causes includes demographic, medical, and obstetrical factors. We will construct IP weights using pooled logistic regression (pooling over time), with continuous variables fit using flexible restricted splines. We will explore the sensitivity to IP weight estimation.[104]

7.10 Dissemination of findings

Study findings will be disseminated through appropriate local channels, including academic and public health research symposia. One or more publications will also be submitted to a peer-reviewed journal. Our study team will plan to publish results whether positive or negative. The study participants' privacy and confidentiality will be strictly maintained in all results dissemination or publication activities.

8.0 Ethical Considerations

The protocol, informed consent documents, and any subsequent modifications will be reviewed and approved by all relevant ethics committees responsible for oversight of the study and maintaining Federal Wide Assurances (FWA) with the Office for Human Research Protections (OHRP) approval. For this study, relevant ethics committees include the University of Zambia Biomedical Research Ethics Committee and the University of North Carolina at Chapel Hill Institutional Review Board.

Participation in this trial will be voluntary. All participants will provide written, informed consent prior to study enrolment. All care and procedures will be conducted according to local standards of routine clinical care. All staff who have contact with participants will receive training on the protection of human research participants prior to conducting any study activities and routinely thereafter. Key staff will also complete Good Clinical Practices training every three years.

8.1 Informed consent

Discussions with prospective participants and informed consent procedures will be conducted in private to protect patient confidentiality. Where possible, a private room will be used to discuss the study and potential participant's eligibility. If a private room is not available, a designated area far enough away from other patients such that they cannot hear the conversation will be used. The study nurse will obtain written informed consent from all participants. The study procedures, risks, and benefits will be discussed and the study nurse will answer all questions prior to obtaining consent. The consent forms will be translated into local languages (Nyanja and Bemba) and back-translated into English to assure accurate translation. All versions of the consent forms will be approved by the relevant ethics committees prior to study initiation. For illiterate participants, a literate impartial witness will be present during the entire consent process to ensure that all of the relevant information has been provided and the participant voluntarily gives consent.

Eligible women who do not wish to participate in this study will continue to receive HIV and antenatal care and treatment according to local clinical standards.

8.2 Potential risks to participants

Investigators will make efforts to minimize risks to participants. It is expected that this study will expose subjects to minimal risks. Side effects and serious adverse events with 17P administration are rare (see ADVERSE EVENTS, below). Side effects may include headache, breast tenderness, nausea and local irritation at the injection site. Women with one or more contraindication to 17P that appear in the package insert will be excluded from the trial, namely: (1) current or history of thrombosis or thromboembolic disorders; (2) known or suspected breast cancer, other hormone-sensitive cancer; or history of these conditions; (3) undiagnosed abnormal vaginal bleeding unrelated to pregnancy; (4) cholestatic jaundice of pregnancy; (5) liver tumor(s), benign or malignant, or active liver disease; (6) uncontrolled hypertension. Serious reactions have not been confirmed in women who do not have these conditions.

Physical risks also include the risk of discomfort, bruising or swelling from venipuncture. The risks that are associated with venipuncture are infrequent and minimized with the use of proper technique. Such risks include (1) bleeding, (2) bruising, or (3) rarely infection at the

site of needle insertion. Individuals may also rarely become faint, in which case symptoms abate after several minutes in a recumbent position. Blood volumes for the study have been calculated to ensure safety. Collection of vaginal and rectal samples may also be associated with some discomfort and mild bleeding. Finally, collection of oral / pharyngeal swabs may cause discomfort; in very few participants, the sensation of gagging may lead to an urge to vomit or cough. Participants will be reassured in all cases that such feelings are transient.

Participation in clinical research includes the risks of loss of confidentiality and discomfort with the personal nature of questions, particularly when discussing HIV infection or sexual behaviors. At each step in the study, we will protect participant privacy and confidentiality to reduce these risks (e.g., consenting participants in a private setting, not including names on case report forms, etc.). Although investigators make every effort to protect participant privacy and confidentiality, it is possible that participant involvement in the study could become known to others, and that social harms may result (i.e. as participants could become known as HIV infected).

The confidentiality of all study records will be safeguarded to the extent legally possible. To maintain participant confidentiality, all laboratory specimens, reports, study data and administrative forms will be identified by a coded number only. All databases will be secured with password-protected access systems, and computer entries will be identified by coded number only. Forms, lists, logbooks, appointment books, and any other listings or data forms that link participant ID numbers to other identifying information will be stored in a separate, locked cabinet. All data analysis will be performed using datasets which have only the study number as a unique identifier. Clinical information with individual identifiers will not be released without the written permission of the participant. We expect these procedures to adequately protect participant confidentiality.

8.3 Potential benefits to participants and others

Individual participants in the 17P arm of the randomized trial may benefit from a reduction in preterm birth risk if the intervention is found to be effective, but it is also possible that there will be no effect. We believe that equipoise exists between the study arms since 17P has not been previously studied in this population of HIV infected women receiving antiretroviral therapy in sub-Saharan Africa, and the exact risk for preterm birth in this population remains unclear. The exact mechanism of action for the potential effect of ART on PTB risk is also unclear, though it is biologically plausible that ART initiation in pregnancy could lead to PTB through an immune activation / inflammatory mechanism. Since 17P is thought to act at least partially through an anti-inflammatory mechanism, it might provide protection for HIV-infected gravidas starting or continuing ART, but this question is yet unanswered.

All participants may benefit from enhanced health education, and close clinical monitoring. Knowledge generated from this study has the potential to inform future clinical trials on the reduction of PTB risk among HIV-infected women, which may enable policymakers worldwide to make informed decisions regarding effective interventions for the prevention of PTB.

At the clinic level, all staff involved in the study will receive refresher training on the national guidelines for the diagnosis and treatment of preterm birth and enhanced training on specimen collection. Increased awareness about screening and treatment for preterm birth may help to improve care overall at UTH and KDC. Additionally, our community

sensitization activities may help to encourage more women to come for antenatal screening earlier in pregnancy, which has a known benefit on maternal and infant outcomes.

In summary, risks to participants in this study are minimal and do not differ significantly from the risks inherent in the local standard of care for HIV-infected pregnant women and their infants, with the exception of the relatively mild side effects of the study drug and the additional risks to participant confidentiality and data privacy. These risks are reasonable in relation to the anticipated benefits if the study drug is found to be effective. Thus, the knowledge generated from this study regarding the potential PTB risk reduction among HIV-infected women is expected to outweigh the risks of participation.

8.4 Inclusion of children, sub-populations, and vulnerable populations

This study focuses specifically on the outcomes of HIV infected pregnant women and their infants and, as such, they must be included in our study population. Prisoners will be excluded as they receive care at separate facilities.

9.0 Timeline

Participants and their infants will be followed from enrollment (prior to 24 weeks gestational age) through 42 days postpartum. We have aligned this follow-up schedule exactly with that of the ongoing GAPPS/PPB cohort. Because that study is already in place at the Zambia site, we anticipate that trial start-up will be quick. We have thus allocated 9 months to start-up activities and 3 months to wind-down activities.

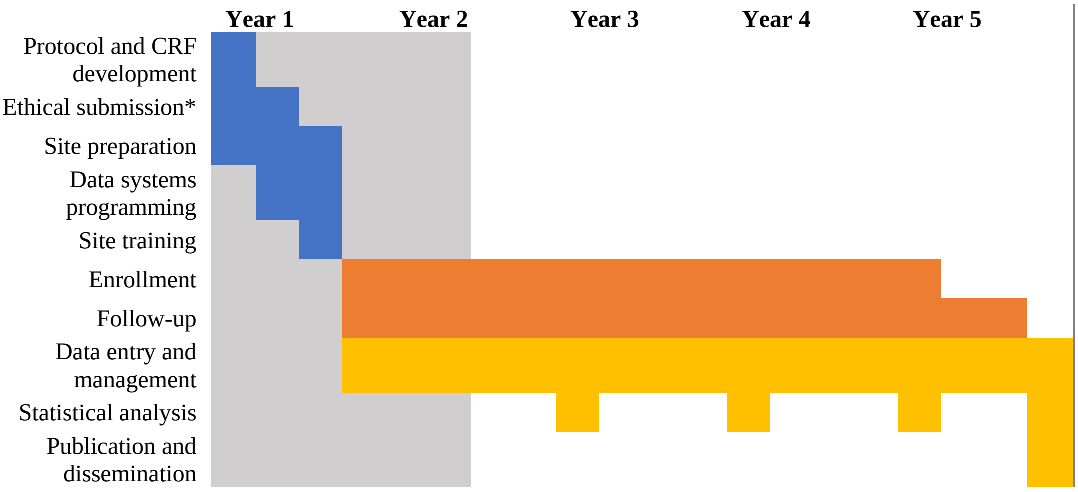

10.0 Budget

The following costs are projected over the five-year study period.

ITEM	ZMW TOTAL	USD TOTAL
Personnel	K 7,820,000	$850,000
Study management	2,622,000	$285,000
Clinic staff	3,266,000	$355,000
Lab staff	1,214,400	$132,000
Pharmacy staff	717,600	$78,000
Equipment – Vehicle	K 368,000	$40,000

Supplies & materials	K 2,024,000	$220,000
Clinic/Office supplies	423,200	$46,000
Lab tests	1,324,800	$144,000
Fuel/Transportation costs	276,000	$30,000
Participant reimbursement	K 828,000	$90,000
Other	K 1,840,000	$200,000
Administrative costs (rent, utilities, internet, communication, shipping, insurance, etc.)	1,058,000	$115,000
Lab maintenance	368,000	$40,000
Community outreach	138,000	$15,000
Regulatory/Translation	110,400	$12,000
Staff Training/Meetings	46,000	$5,000
Analysis/Dissemination costs	119,600	$13,000
TOTAL	K 12,880,000	$1,400,000

11.0 Reference List

1. Blencowe H, Cousens S, Oestergaard MZ, et al. National, regional, and worldwide estimates of preterm birth rates in the year 2010 with time trends since 1990 for selected countries: a systematic analysis and implications. *The Lancet.* 2012;379(9832):2162-2172.
2. Brocklehurst P. The Association between maternal HIV infection and perinatal outcome: a systematic review of the literature and meta-analysis. *Br J Obstet Gynaecol.*105:836-848.
3. Wedi CO, Kirtley S, Hopewell S, Corrigan R, Kennedy SH, Hemelaar J. Perinatal outcomes associated with maternal HIV infection: a systematic review and meta-analysis. *Lancet HIV.* 2016;3(1):e33-48.
4. Fowler MG, Qin M., Fiscus S.A., Currier J.S., Makanani B,. Martinson F., Chipato T., Browning R., Shapiro D., Mofenson L. PROMISE: Efficacy and safety of 2 strategies to prevent perinatal HIV transmission. Conference on Retroviruses and Opportunistic Infections; February 23-24, 2015; Seattle.
5. Dodd J, Jones L, Flenady V, Cincotta R, Crowther C. Prenatal administration of progesterone for preventing preterm birth in women considered to be at risk of preterm birth. *Cochrane Database of Systematic Reviews.* 2013(7).
6. Rouse DJ, Caritis SN, Peaceman AM, et al. A trial of 17 alpha-hydroxyprogesterone caproate to prevent prematurity in twins. *N Engl J Med.* 2007;357(5):454-461.
7. Grobman WA, Thom EA, Spong CY, et al. 17 alpha-hydroxyprogesterone caproate to prevent prematurity in nulliparas with cervical length less than 30 mm. *Am J Obstet Gynecol.* 2012;207(5):390 e391-398.
8. Caritis SN, Rouse DJ, Peaceman AM, et al. Prevention of preterm birth in triplets using 17 alpha-hydroxyprogesterone caproate: a randomized controlled trial. *Obstet Gynecol.* 2009;113(2 Pt 1):285-292.
9. Meis PJ, Klebanoff M, Thom E, et al. Prevention of recurrent preterm delivery by 17 alpha-hydroxyprogesterone caproate. *N Engl J Med.* 2003;348(24):2379-2385.
10. Saghafi N, Khadem N, Mohajeri T, Shakeri MT. Efficacy of 17alpha-hydroxyprogesterone caproate in prevention of preterm delivery. *J Obstet Gynaecol Res.* 2011;37(10):1342-1345.
11. Johnson J, Austin K, Jones G, Davis G, King T. Efficacy of 17 alpha-hydroxyprogesterone caproate in the prevention of premature labor. *New England Journal of Medicine* 1975;293(14):675-680.
12. Ibrahim M, Mohamed Ramy AR, Younis MA-F. Progesterone supplementation for prevention of preterm labor: A randomized controlled trial. *Middle East Fertility Society Journal.* 2010;15(1):39-41.
13. Rozenberg P, Chauveaud A, Deruelle P, et al. Prevention of preterm delivery after successful tocolysis in preterm labor by 17 alpha-hydroxyprogesterone caproate: a randomized controlled trial. *Am J Obstet Gynecol.* 2012;206(3):206 e201-209.
14. Senat MV, Porcher R, Winer N, et al. Prevention of preterm delivery by 17 alpha-hydroxyprogesterone caproate in asymptomatic twin pregnancies with a short cervix: a randomized controlled trial. *Am J Obstet Gynecol.* 2013;208(3):194 e191-198.
15. Combs CA, Garite T, Maurel K, Das A, Porto M, Obstetrix Collaborative Research N. 17-hydroxyprogesterone caproate for twin pregnancy: a double-blind, randomized clinical trial. *Am J Obstet Gynecol.* 2011;204(3):221 e221-228.
16. Awwad J, Usta IM, Ghazeeri G, et al. A randomised controlled double-blind clinical trial of 17-hydroxyprogesterone caproate for the prevention of preterm birth in twin

gestation (PROGESTWIN): evidence for reduced neonatal morbidity. *BJOG.* 2015;122(1):71-79.
17. Briery CM, Klauser CK, Martin RW, Magann EF, Chauhan SP, Morrison JC. The use of 17-hydroxy progesterone in women with arrested preterm labor: a randomized clinical trial. *J Matern Fetal Neonatal Med.* 2014;27(18):1892-1896.
18. Lotfalizadeh M, Ghomian N, Reyhani A. The Effects of Progesterone Therapy on the Gestation Length and Reduction of Neonatal Complications in Patients who had Received Tocolytic Therapy for Acute Phase of Preterm Labor. *Iran Red Crescent Med J.* 2013;15(10):e7947.
19. Townsend CL, Tookey PA, Newell ML, Cortina-Borja M. Antiretroviral therapy in pregnancy: balancing the risk of preterm delivery with prevention of mother-to-child HIV transmission. *Antivir Ther.* 2010;15(5):775-783.
20. Rinaldi SF, Hutchinson JL, Rossi AG, Norman JE. Anti-inflammatory mediators as physiological and pharmacological regulators of parturition. *Expert Rev Clin Immunol.* 2011;7(5):675-696.
21. March of Dimes P, Save the Children, WHO. *Born too Soon: the Global Action Report on Preterm Birth.* Geneva: WHO;2012.
22. Hack M, Taylor HG, Drotar D, et al. Chronic conditions, functional limitations, and special health care needs of school-aged children born with extremely low-birth-weight in the 1990s. *JAMA.* 2005;294(3):318-325.
23. Barros FC, Bhutta ZA, Batra M, et al. Global report on preterm birth and stillbirth (3 of 7): evidence for effectiveness of interventions. *BMC Pregnancy Childbirth.* 2010;10 Suppl 1:S3.
24. Joint United Nations Programme on HIV/AIDS. The Gap Report. 2014; http://www.unaids.org/sites/default/files/media_asset/UNAIDS_Gap_report_en.pdf. Accessed March 14, 2016.
25. Chen JY, Ribaudo HJ, Souda S, et al. Highly active antiretroviral therapy and adverse birth outcomes among HIV-infected women in Botswana. *J Infect Dis.* 2012;206(11):1695-1705.
26. Powis KM, Kitch D, Ogwu A, et al. Increased risk of preterm delivery among HIV-infected women randomized to protease versus nucleoside reverse transcriptase inhibitor-based HAART during pregnancy. *J Infect Dis.* 2011;204(4):506-514.
27. Cotter AM, Garcia AG, Duthely ML, Luke B, O'Sullivan MJ. Is antiretroviral therapy during pregnancy associated with an increased risk of preterm delivery, low birth weight, or stillbirth? *The Journal of infectious diseases.* 2006;193(9):1195-1201.
28. Li N, Sando MM, Spiegelman D, et al. Antiretroviral Therapy in Relation to Birth Outcomes among HIV-infected Women: A Cohort Study. *J Infect Dis.* 2016;213(7):1057-1064.
29. van der Merwe K, Hoffman R, Black V, Chersich M, Coovadia A, Rees H. Birth outcomes in South African women receiving highly active antiretroviral therapy: a retrospective observational study. *J Int AIDS Soc.* 2011;14:42.
30. Townsend CL, Cortina-Borja M, Peckham CS, Tookey PA. Antiretroviral therapy and premature delivery in diagnosed HIV-infected women in the United Kingdom and Ireland. *AIDS.* 2007;21(8):1019-1026.
31. Darak S, Darak T, Kulkarni S, et al. Effect of highly active antiretroviral treatment (HAART) during pregnancy on pregnancy outcomes: experiences from a PMTCT program in western India. *AIDS Patient Care STDS.* 2013;27(3):163-170.
32. Townsend C, Schulte J, Thorne C, et al. Antiretroviral therapy and preterm delivery-a pooled analysis of data from the United States and Europe. *BJOG.* 2010;117(11):1399-1410.

33. Machado ES, Hofer CB, Costa TT, et al. Pregnancy outcome in women infected with HIV-1 receiving combination antiretroviral therapy before versus after conception. *Sex Transm Infect.* 2009;85(2):82-87.
34. Koss CA, Natureeba P, Plenty A, et al. Risk factors for preterm birth among HIV-infected pregnant Ugandan women randomized to lopinavir/ritonavir- or efavirenz-based antiretroviral therapy. *J Acquir Immune Defic Syndr.* 2014;67(2):128-135.
35. Lorenzi P, Spicher VM, Laubereau B, et al. Antiretroviral therapies in pregnancy: maternal, fetal and neonatal effects. Swiss HIV Cohort Study, the Swiss Collaborative HIV and Pregnancy Study, and the Swiss Neonatal HIV Study. *AIDS.* 1998;12(18):F241-247.
36. Martin F, Taylor GP. Increased rates of preterm delivery are associated with the initiation of highly active antiretrovial therapy during pregnancy: a single-center cohort study. *J Infect Dis.* 2007;196(4):558-561.
37. European Collaborative S, Swiss M, Child HIVCS. Combination antiretroviral therapy and duration of pregnancy. *AIDS.* 2000;14(18):2913-2920.
38. Sibiude J, Warszawski J, Tubiana R, et al. Premature delivery in HIV-infected women starting protease inhibitor therapy during pregnancy: role of the ritonavir boost? *Clin Infect Dis.* 2012;54(9):1348-1360.
39. Thorne C, Patel D, Newell ML. Increased risk of adverse pregnancy outcomes in HIV-infected women treated with highly active antiretroviral therapy in Europe. *AIDS.* 2004;18(17):2337-2339.
40. Society for Maternal-Fetal Medicine Publications Committee waoVB. Progesterone and preterm birth prevention: translating clinical trials data into clinical practice. *Am J Obstet Gynecol.* 2012;206(5):376-386.
41. Iams JD, Romero R, Culhane JF, Goldenberg RL. Primary, secondary, and tertiary interventions to reduce the morbidity and mortality of preterm birth. *The Lancet.* 2008;371(9607):164-175.
42. Rubens CE, Sadovsky Y, Muglia L, Gravett MG, Lackritz E, Gravett C. Prevention of preterm birth: Harnessing science to address the global epidemic. *Sci Transl Med.* 2014;6(262):262sr265.
43. Luo G, Abrahams VM, Tadesse S, et al. Progesterone inhibits basal and TNF-alpha-induced apoptosis in fetal membranes: a novel mechanism to explain progesterone-mediated prevention of preterm birth. *Reprod Sci.* 2010;17(6):532-539.
44. Shields AD, Wright J, Paonessa DJ, et al. Progesterone modulation of inflammatory cytokine production in a fetoplacental artery explant model. *Am J Obstet Gynecol.* 2005;193(3 Pt 2):1144-1148.
45. Gotkin JL, Celver J, McNutt P, et al. Progesterone reduces lipopolysaccharide induced interleukin-6 secretion in fetoplacental chorionic arteries, fractionated cord blood, and maternal mononuclear cells. *Am J Obstet Gynecol.* 2006;195(4):1015-1019.
46. Schwartz N, Xue X, Elovitz MA, Dowling O, Metz CN. Progesterone suppresses the fetal inflammatory response ex vivo. *Am J Obstet Gynecol.* 2009;201(2):211 e211-219.
47. Wolf JP, Sinosich M, Anderson TL, Ulmann A, Baulieu EE, Hodgen GD. Progesterone antagonist (RU 486) for cervical dilation, labor induction, and delivery in monkeys: effectiveness in combination with oxytocin. *Am J Obstet Gynecol.* 1989;160(1):45-47.
48. Elovitz MA, Mrinalini C. The use of progestational agents for preterm birth: lessons from a mouse model. *Am J Obstet Gynecol.* 2006;195(4):1004-1010.

49. Mazor M, Hershkowitz R, Ghezzi F, et al. Maternal plasma and amniotic fluid 17 beta-estradiol, progesterone and cortisol concentrations in women with successfully and unsuccessfully treated preterm labor. *Arch Gynecol Obstet.* 1996;258(2):89-96.
50. Caritis SN, Venkataramanan R, Thom E, et al. Relationship between 17-alpha hydroxyprogesterone caproate concentration and spontaneous preterm birth. *Am J Obstet Gynecol.* 2014;210(2):128 e121-126.
51. Appay V, Sauce D. Immune activation and inflammation in HIV-1 infection: causes and consequences. *J Pathol.* 2008;214(2):231-241.
52. Spencer LY, Christiansen S, Wang CH, et al. Systemic Immune Activation and HIV Shedding in the Female Genital Tract. *J Acquir Immune Defic Syndr.* 2016;71(2):155-162.
53. Neely MN, Benning L, Xu J, et al. Cervical shedding of HIV-1 RNA among women with low levels of viremia while receiving highly active antiretroviral therapy. *J Acquir Immune Defic Syndr.* 2007;44(1):38-42.
54. Cu-Uvin S, DeLong AK, Venkatesh KK, et al. Genital tract HIV-1 RNA shedding among women with below detectable plasma viral load. *AIDS.* 2010;24(16):2489-2497.
55. Ondoa P, Gautam R, Rusine J, et al. Twelve-Month Antiretroviral Therapy Suppresses Plasma and Genital Viral Loads but Fails to Alter Genital Levels of Cytokines, in a Cohort of HIV-Infected Rwandan Women. *PLoS One.* 2015;10(5):e0127201.
56. Cohen CR, Lingappa JR, Baeten JM, et al. Bacterial vaginosis associated with increased risk of female-to-male HIV-1 transmission: a prospective cohort analysis among African couples. *PLoS Med.* 2012;9(6):e1001251.
57. Martin HL, Richardson BA, Nyange PM, et al. Vaginal lactobacilli, microbial flora, and risk of human immunodeficiency virus type 1 and sexually transmitted disease acquisition. *J Infect Dis.* 1999;180(6):1863-1868.
58. Myer L, Denny L, Telerant R, Souza M, Wright TC, Jr., Kuhn L. Bacterial vaginosis and susceptibility to HIV infection in South African women: a nested case-control study. *J Infect Dis.* 2005;192(8):1372-1380.
59. Bretelle F, Rozenberg P, Pascal A, et al. High Atopobium vaginae and Gardnerella vaginalis vaginal loads are associated with preterm birth. *Clin Infect Dis.* 2015;60(6):860-867.
60. Meis PJ, Goldenberg RL, Mercer B, et al. The preterm prediction study: significance of vaginal infections. National Institute of Child Health and Human Development Maternal-Fetal Medicine Units Network. *Am J Obstet Gynecol.* 1995;173(4):1231-1235.
61. Warren D, Klein RS, Sobel J, et al. A multicenter study of bacterial vaginosis in women with or at risk for human immunodeficiency virus infection. *Infect Dis Obstet Gynecol.* 2001;9(3):133-141.
62. Gianella S, Redd AD, Grabowski MK, et al. Vaginal Cytomegalovirus Shedding Before and After Initiation of Antiretroviral Therapy in Rakai, Uganda. *J Infect Dis.* 2015;212(6):899-903.
63. Tobian AA, Grabowski MK, Serwadda D, et al. Reactivation of herpes simplex virus type 2 after initiation of antiretroviral therapy. *J Infect Dis.* 2013;208(5):839-846.
64. Akbari S, Birjandi M, Mohtasham N. Evaluation of the effect of progesterone on prevention of preterm delivery and its complications. *Scientific Journal of Kurdistan University of Medical Sciences.* 2009;14(3):9-11.

65. Cetingoz E, Cam C, Sakalli M, Karateke A, Celik C, Sancak A. Progesterone effects on preterm birth in high-risk pregnancies: a randomized placebo-controlled trial. *Arch Gynecol Obstet.* 2011;283(3):423-429.
66. da Fonseca EB, Bittar RE, Carvalho MH, Zugaib M. Prophylactic administration of progesterone by vaginal suppository to reduce the incidence of spontaneous preterm birth in women at increased risk: a randomized placebo-controlled double-blind study. *Am J Obstet Gynecol.* 2003;188(2):419-424.
67. Majhi P, Bagga R, Kalra J, Sharma M. Intravaginal use of natural micronised progesterone to prevent pre-term birth: a randomised trial in India. *J Obstet Gynaecol.* 2009;29(6):493-498.
68. O'Brien JM, Adair CD, Lewis DF, et al. Progesterone vaginal gel for the reduction of recurrent preterm birth: primary results from a randomized, double-blind, placebo-controlled trial. *Ultrasound Obstet Gynecol.* 2007;30(5):687-696.
69. Gotsch F, Romero R, Erez O, et al. The preterm parturition syndrome and its implications for understanding the biology, risk assessment, diagnosis, treatment and prevention of preterm birth. *J Matern Fetal Neonatal Med.* 2009;22 Suppl 2:5-23.
70. Myatt L, Eschenbach DA, Lye SJ, et al. A standardized template for clinical studies in preterm birth. *Reprod Sci.* 2012;19(5):474-482.
71. Revesz C, Chappel C, Gaudry R. Masculinization of Female Fetuses in the Rat by Progestational Compounds. *Obstetrical & Gynecological Survey.* 1960;15(4):543.
72. Suchowsky GK, Turolla E, Arcari G. Studies of the so-called virilizing effects of steroids in female rat fetuses. *Endocrinology.* 1967;80(2):255-262.
73. Grumbach MM, Ducharme JR, Moloshok RE. On the fetal masculinizing action of certain oral progestins. *J Clin Endocrinol Metab.* 1959;19:1369-1380.
74. Wilkins L, Jones HW, Jr., Holman GH, Stempfel RS, Jr. Masculinization of the female fetus associated with administration of oral and intramuscular progestins during gestation: non-adrenal female pseudohermaphrodism. *J Clin Endocrinol Metab.* 1958;18(6):559-585.
75. Johnstone EE, Franklin RR. Assay of Progestins for Fetal Virilizing Properties Using the Mouse. *Obstet Gynecol.* 1964;23:359-362.
76. Suchowsky G, Junkmann K. [Research on the maintenance of pregnancy by 17 alpha-hydroxyprogesterone caproate in the castrated pregnant rabbit]. *Acta Endocrinol (Copenh).* 1958;28(2):129-131.
77. Junkmann K. [Estrogens with prolonged action]. *Naunyn Schmiedebergs Arch Exp Pathol Pharmakol.* 1953;220(5):358-364.
78. Kessler WB, Borman A. Some biological activities of certain progestogens. I. 17 alpha-Hydroxyprogesterone 17-n-caproate. *Ann N Y Acad Sci.* 1958;71(5):486-493.
79. Courtney KD, Valerio DA. Teratology in the Macaca mulatta. *Teratology.* 1968;1(2):163-172.
80. Seegmiller RE, Nelson GW, Johnson CK. Evaluation of the teratogenic potential of delalutin (17 alpha-hydroxyprogesterone caproate) in mice. *Teratology.* 1983;28(2):201-208.
81. Carbone JP, Brent RL. Genital and nongenital teratogenesis of prenatal progestogen therapy: the effects of 17 alpha-hydroxyprogesterone caproate on embryonic and fetal development and endochondral ossification in the C57B1/6J mouse. *Am J Obstet Gynecol.* 1993;169(5):1292-1298.
82. da Fonseca EB, Bittar RE, Carvalho MHB, Zugaib M. Prophylactic administration of progesterone by vaginal suppository to reduce the incidence of spontaneous preterm birth in women at increased risk: A randomized placebo-controlled double-blind study. *American Journal of Obstetrics and Gynecology.* 2003;188(2):419-424.

83. Hassan SS, Romero R, Vidyadhari D, et al. Vaginal progesterone reduces the rate of preterm birth in women with a sonographic short cervix: a multicenter, randomized, double-blind, placebo-controlled trial. *Ultrasound Obstet Gynecol.* 2011;38(1):18-31.
84. Resseguie LJ, Hick JF, Bruen JA, Noller KL, O'Fallon WM, Kurland LT. Congenital malformations among offspring exposed in utero to progestins, Olmsted County, Minnesota, 1936-1974. *Fertil Steril.* 1985;43(4):514-519.
85. Raman-Wilms L, Tseng AL, Wighardt S, Einarson TR, Koren G. Fetal genital effects of first-trimester sex hormone exposure: a meta-analysis. *Obstet Gynecol.* 1995;85(1):141-149.
86. Schuit E, Stock S, Rode L, et al. Effectiveness of progestogens to improve perinatal outcome in twin pregnancies: an individual participant data meta-analysis. *BJOG.* 2015;122(1):27-37.
87. Schardein JL. Congenital abnormalities and hormones during pregnancy: a clinical review. *Teratology.* 1980;22(3):251-270.
88. Katz Z, Lancet M, Skornik J, Chemke J, Mogilner BM, Klinberg M. Teratogenicity of progestogens given during the first trimester of pregnancy. *Obstet Gynecol.* 1985;65(6):775-780.
89. Makena. Makena Prescribing Information [package insert]. Available at http://www.makena.com/pdf/makena_pi.pdf. . Accessed 5 May 2015.
90. Iams JD, Goldenberg RL, Meis PJ, et al. The length of the cervix and the risk of spontaneous premature delivery. National Institute of Child Health and Human Development Maternal Fetal Medicine Unit Network. *N Engl J Med.* 1996;334(9):567-572.
91. Chi BH, Vwalika B, Killam WP, et al. Implementation of the Zambia electronic perinatal record system for comprehensive prenatal and delivery care. *Int J Gynaecol Obstet.* 2011;113(2):131-136.
92. Schultz K, Grimes D. Generation of allocation sequences in randomised trials: chance, not choice. *Lancet.* 2002;359:515-519.
93. Stringer EM, Vwalika B, Killam WP, et al. Determinants of stillbirth in Zambia. *Obstet Gynecol.* 2011;117(5):1151-1159.
94. Feresu SA, Harlow SD, Welch K, Gillespie BW. Incidence of and socio-demographic risk factors for stillbirth, preterm birth and low birthweight among Zimbabwean women. *Paediatr Perinat Epidemiol.* 2004;18(2):154-163.
95. Friis H, Gomo E, Nyazema N, et al. Effect of multimicronutrient supplementation on gestational length and birth size: a randomized, placebo-controlled, double-blind effectiveness trial in Zimbabwe. *Am J Clin Nutr.* 2004;80(1):178-184.
96. Ticconi C, Arpino C, Longo B, Mapfumo M. Prevalence and risk factors for low birth weight in Northern Zimbabwe. *Int J Gynaecol Obstet.* 2005;88(2):146-147.
97. Papageorghiou AT, Kemp B, Stones W, et al. Ultrasound-based gestational-age estimation in late pregnancy. *Ultrasound Obstet Gynecol.* 2016;48(6):719-726.
98. Papageorghiou AT, Kennedy SH, Salomon LJ, et al. International standards for early fetal size and pregnancy dating based on ultrasound measurement of crown-rump length in the first trimester of pregnancy. *Ultrasound Obstet Gynecol.* 2014;44(6):641-648.
99. Hadlock FP, Shah YP, Kanon DJ, Lindsey JV. Fetal crown-rump length: reevaluation of relation to menstrual age (5-18 weeks) with high-resolution real-time US. *Radiology.* 1992;182(2):501-505.
100. Robins JM, Hernan MA, Brumback B. Marginal structural models and causal inference in epidemiology. *Epidemiology.* 2000;11(5):550-560.

101. Cole SR, Chu H. Effect of acyclovir on herpetic ocular recurrence using a structural nested model. *Contemp Clin Trials.* 2005;26(3):300-310.
102. Cook NR, Cole SR, Buring JE. Aspirin in the primary prevention of cardiovascular disease in the Women's Health Study: effect of noncompliance. *Eur J Epidemiol.* 2012;27(6):431-438.
103. Cain LE, Cole SR. Inverse probability-of-censoring weights for the correction of time-varying noncompliance in the effect of randomized highly active antiretroviral therapy on incident AIDS or death. *Stat Med.* 2009;28(12):1725-1738.
104. Cole SR, Hernan MA. Constructing inverse probability weights for marginal structural models. *Am J Epidemiol.* 2008;168(6):656-664.
