# Supplementary material for: Reducing Bias in Estimates of Per Protocol Treatment Effects: A Secondary Analysis of a Randomized Clinical Trial
Source: JAMA Netw Open. 2023 Jul 26;6(7):e2325907. doi: 10.1001/jamanetworkopen.2023.25907 (PMC10372700; doi:10.1001/jamanetworkopen.2023.25907)
Supplement: Supplement 2. — eReferences [file jamanetwopen-e2325907-s002.pdf]

Supplemental Online Content

Cole SR, Edwards JK, Zivich PN, Shook-Sa BE, Hudgens MG, Stringer JSA. Reducing bias in estimates of per protocol treatment effects: a secondary analysis of a randomized clinical trial. *JAMA Netw Open*. 2023;6(7):e2325907. doi:10.1001/jamanetworkopen.2023.25907

eReferences

This supplemental material has been provided by the authors to give readers additional information about their work.

© 2023 Cole SR et al. *JAMA Network Open.*

eReferences

e1. Goldenberg NA, Kittelson JM, Abshire TC, et al. Effect of Anticoagulant Therapy for 6 Weeks vs 3 Months on Recurrence and Bleeding Events in Patients Younger Than 21 Years of Age With Provoked Venous Thromboembolism: The Kids-DOTT Randomized Clinical Trial. *JAMA.* 2022;327(2):129-137.

e2. Grillot N, Lebuffe G, Huet O, et al. Effect of Remifentanil vs Neuromuscular Blockers During Rapid Sequence Intubation on Successful Intubation Without Major Complications Among Patients at Risk of Aspiration: A Randomized Clinical Trial. *JAMA.* 2023;329(1):28-38.

e3. Nyang'wa BT, Berry C, Kazounis E, et al. A 24-Week, All-Oral Regimen for Rifampin-Resistant Tuberculosis. *N Engl J Med.* 2022;387(25):2331-2343.

e4. Reis G, Silva E, Silva DCM, et al. Effect of Early Treatment with Ivermectin among Patients with Covid-19. *N Engl J Med.* 2022;386(18):1721-1731.

e5. Turkova A, Wills GH, Wobudeya E, et al. Shorter Treatment for Nonsevere Tuberculosis in African and Indian Children. *N Engl J Med.* 2022;386(10):911-922.

e6. van der Vaart LR, Vollebregt A, Milani AL, et al. Effect of Pessary vs Surgery on Patient-Reported Improvement in Patients With Symptomatic Pelvic Organ Prolapse: A Randomized Clinical Trial. *JAMA.* 2022;328(23):2312-2323.

e7. Varghese GM, Dayanand D, Gunasekaran K, et al. Intravenous Doxycycline, Azithromycin, or Both for Severe Scrub Typhus. *N Engl J Med.* 2023;388(9):792-803.

© 2023 Cole SR et al. *JAMA Network Open.*
