# Supplementary material for: Reducing Bias in Estimates of Per Protocol Treatment Effects: A Secondary Analysis of a Randomized Clinical Trial
Source: JAMA Netw Open. 2023 Jul 26;6(7):e2325907. doi: 10.1001/jamanetworkopen.2023.25907 (PMC10372700; doi:10.1001/jamanetworkopen.2023.25907)
Supplement: Supplement 3. — Data Sharing Statement [file jamanetwopen-e2325907-s003.pdf]

Data Sharing Statement

Cole. Reducing Bias in Estimates of Per Protocol Treatment Effects. *JAMA Netw Open*. Published July 26, 2023. doi:10.1001/jamanetworkopen.2023.25907

Data
Data available: Yes
Data types: Deidentified participant data
How to access data: https://github.com/CIRL-UNC/StandardizedPerProtocol
When available: With publication

Supporting Documents
Document types: Statistical/analytic code
How to access documents: https://github.com/CIRL-UNC/StandardizedPerProtocol
When available: With publication

Additional Information
Who can access the data: Anyone
Types of analyses: Any purpose
Mechanisms of data availability: Without investigator support
